# Supplementary figures and images for: Phospho-RNA-Seq Highlights Specific Small RNA Profiles in Plasma Extracellular Vesicles
Source: Int J Mol Sci. 2023 Jul 19;24(14):11653. doi: 10.3390/ijms241411653 (PMC10380198; doi:10.3390/ijms241411653)

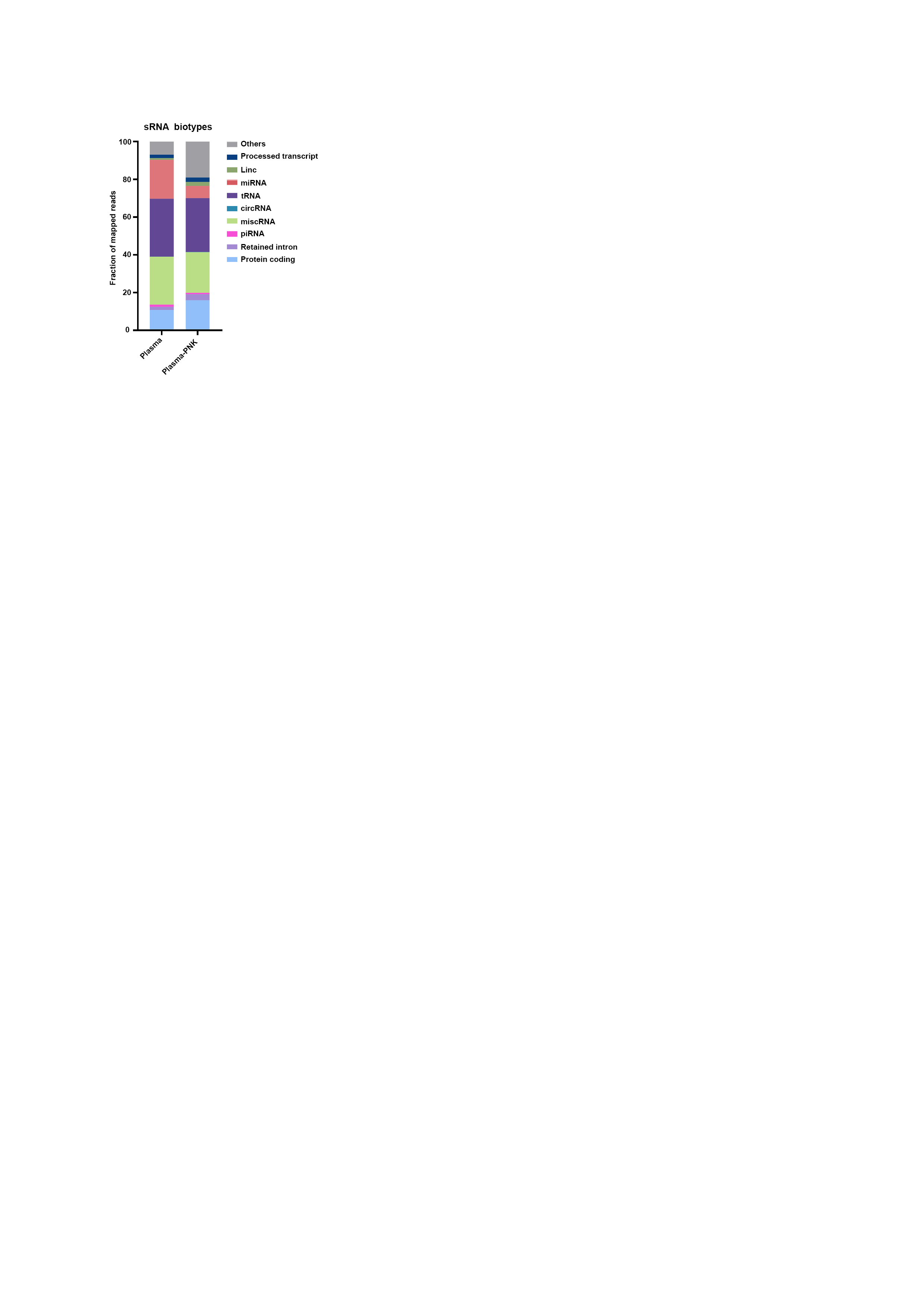

Supplement: Supplementary file 1 [file ijms-24-11653-s001.zip › ijms-2441202-supplementary/SupplementaryFigure_S1.tif]

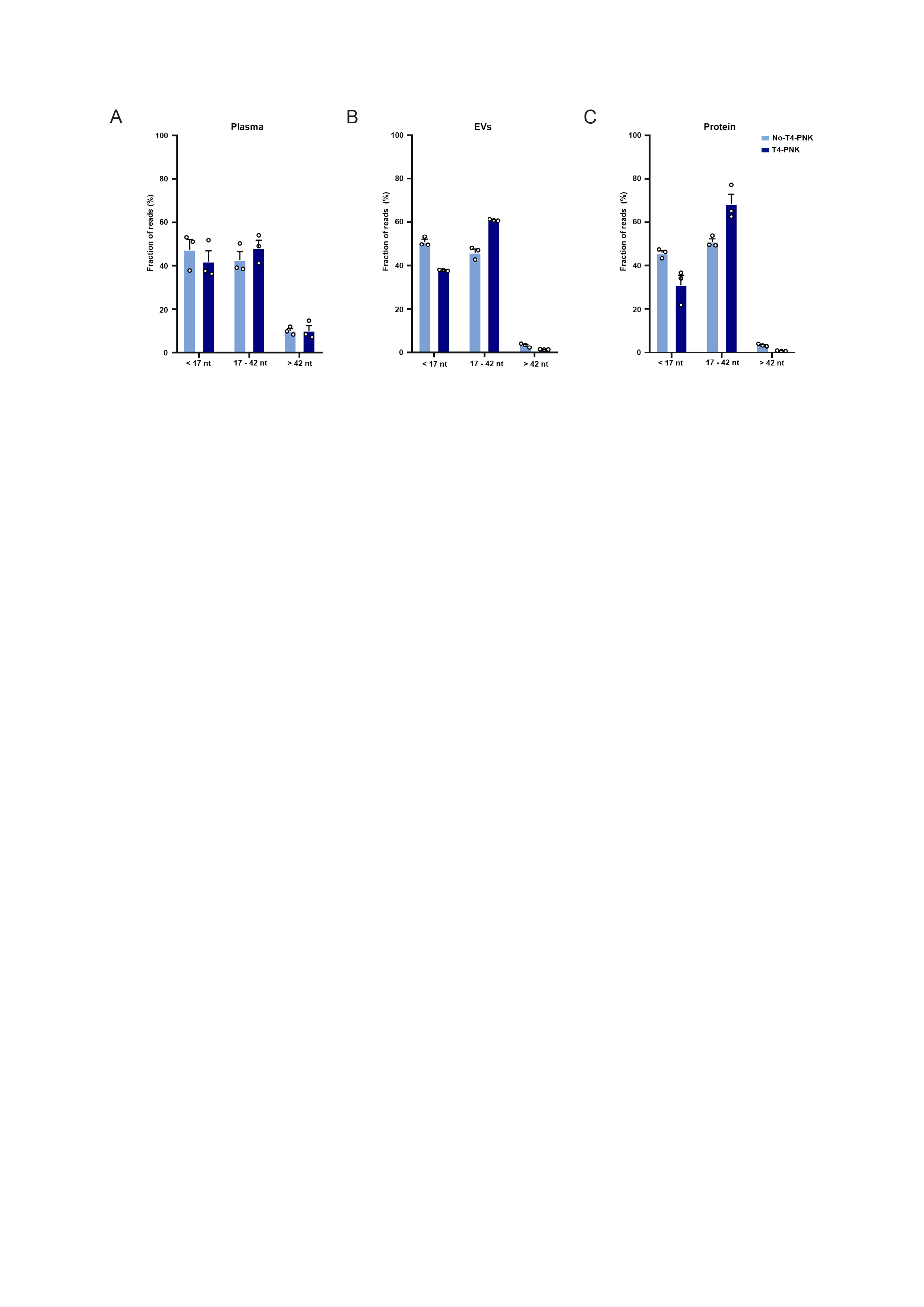

Supplement: Supplementary file 1 [file ijms-24-11653-s001.zip › ijms-2441202-supplementary/SupplementaryFigure_S2.tif]

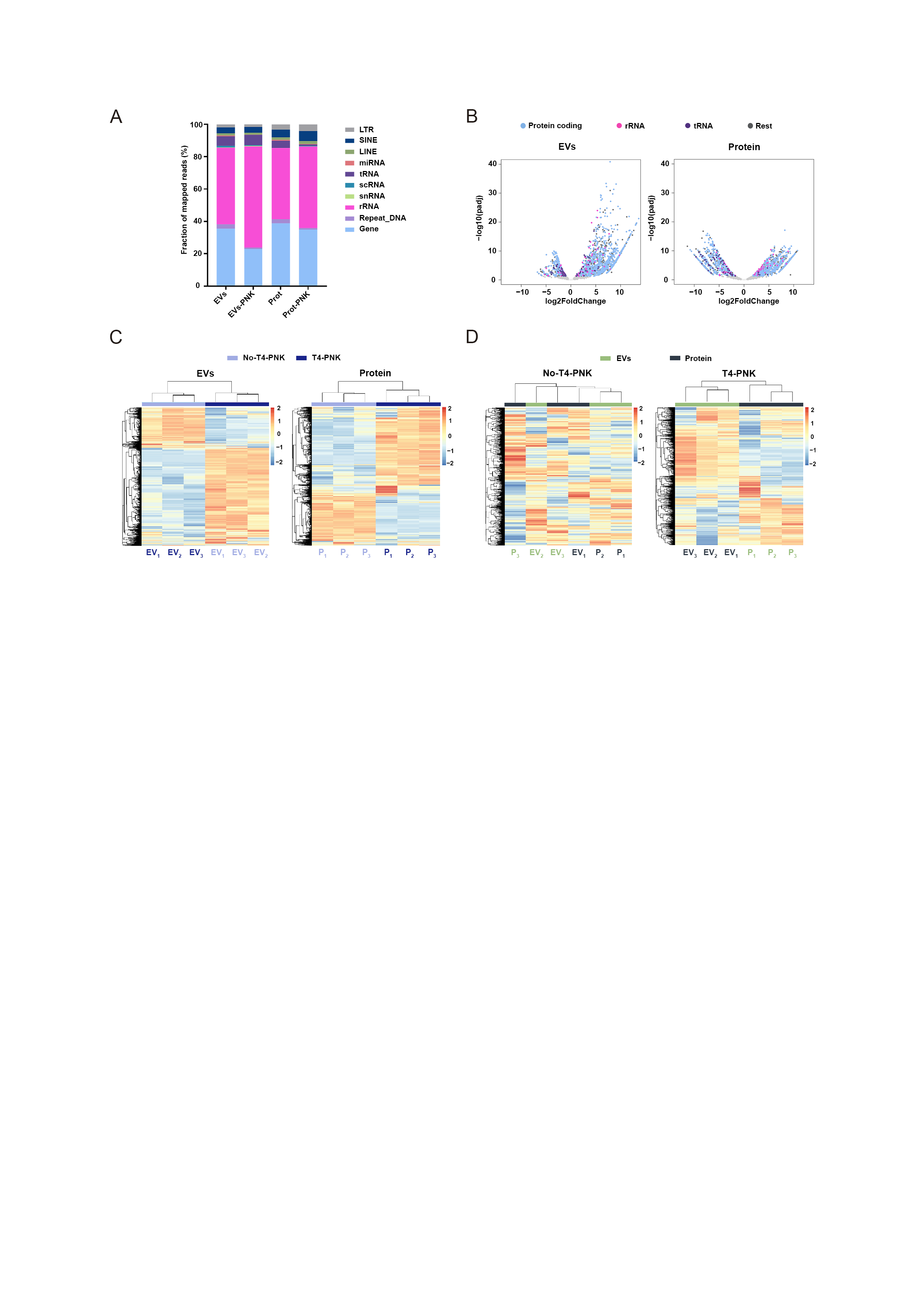

Supplement: Supplementary file 1 [file ijms-24-11653-s001.zip › ijms-2441202-supplementary/SupplementaryFigure_S3_v2.tif]
